# What must be done to tackle vaccine hesitancy and barriers to COVID-19 vaccination in migrants?

**DOI:** 10.1093/jtm/taab048

**Published:** 2021-03-26

**Authors:** Alison F Crawshaw, Anna Deal, Kieran Rustage, Alice S Forster, Ines Campos-Matos, Tushna Vandrevala, Andrea Würz, Anastasia Pharris, Jonathan E Suk, John Kinsman, Charlotte Deogan, Anna Miller, Silvia Declich, Chris Greenaway, Teymur Noori, Sally Hargreaves

**Affiliations:** Migrant Health Research Group, Institute for Infection and Immunity, St George’s, University of London, SW17 0RE London, UK; Migrant Health Research Group, Institute for Infection and Immunity, St George’s, University of London, SW17 0RE London, UK; Faculty of Public Health and Policy, London School of Hygiene and Tropical Medicine, WC1E 7HT London, UK; Migrant Health Research Group, Institute for Infection and Immunity, St George’s, University of London, SW17 0RE London, UK; Department of Behavioural Science and Health, University College London, WC1E 6BT London, UK; Health Improvement Division, Public Health England, SE1 8UG London, UK and UCL Collaborative Centre for Inclusion Health, University College London, WC1E 6BT London, UK; Department of Psychology, Kingston University London, Kingston KT2 7LB, UK; Disease Programmes (AW, AP, JK, CD, TN); Public Health Functions (JES), European Centre for Disease Prevention and Control, Stockholm SE-Solna, Sweden; Disease Programmes (AW, AP, JK, CD, TN); Public Health Functions (JES), European Centre for Disease Prevention and Control, Stockholm SE-Solna, Sweden; Disease Programmes (AW, AP, JK, CD, TN); Public Health Functions (JES), European Centre for Disease Prevention and Control, Stockholm SE-Solna, Sweden; Disease Programmes (AW, AP, JK, CD, TN); Public Health Functions (JES), European Centre for Disease Prevention and Control, Stockholm SE-Solna, Sweden; Disease Programmes (AW, AP, JK, CD, TN); Public Health Functions (JES), European Centre for Disease Prevention and Control, Stockholm SE-Solna, Sweden; Policy & Advocacy Division, Doctors of the World UK, part of the Médicins du Monde network, E14 5AA London, UK; National Centre for Global Health, Istituto Superiore di Sanità, 00161 Roma RM, Italy; Department of Medicine, McGill University Montreal, H3A 1A1 Quebec, Canada; Disease Programmes (AW, AP, JK, CD, TN); Public Health Functions (JES), European Centre for Disease Prevention and Control, Stockholm SE-Solna, Sweden; Migrant Health Research Group, Institute for Infection and Immunity, St George’s, University of London, SW17 0RE London, UK

**Keywords:** Migrants, COVID-19, vaccination

## Abstract

Migrants have been disproportionately impacted by COVID-19 and emerging evidence suggests they may face barriers to COVID-19 vaccination. Participatory approaches and engagement strategies are urgently needed to strengthen uptake, alongside innovative delivery mechanisms and sharing of best practice, to ensure migrants are better consider within countries’ existing vaccine priority structures.

Emerging evidence from high-income countries shows that ethnic minority populations, which include diverse groups of migrants, may be more reluctant than others to accept a vaccine against coronavirus disease 2019 (COVID-19).^1–3^ One UK study (*n* = 11 708), for example, found COVID-19 vaccine hesitancy—a reluctance or refusal to vaccinate despite the availability of vaccines—was the highest in Black ethnic groups, with 72% reportedly unlikely or very unlikely to be vaccinated.^3^ Vaccine hesitancy poses a threat for COVID-19 and control of all vaccine-preventable diseases, and was considered one of WHO’s (World Health Organization) top 10 global health threats in 2019. Particular concerns are being raised around hesitancy and other barriers to vaccination specifically in migrant groups (foreign-born nationals), which are currently poorly elucidated. In addition, large numbers of more recently arrived migrants remain outside of health systems in many countries, due to, for example, lack of legal entitlement, and thus risk being excluded from vaccine roll-out. This includes undocumented migrants, asylum seekers and refugees, those residing in camps, detention centres and other high-risk settings, alongside specific communities such as the Roma. It is important to act quickly upon these issues, because migrants make up sizeable populations and workforces in many high-income countries and have experienced adverse clinical outcomes, including being disproportionately represented in COVID-19 cases and deaths,^4^^,^^5^ and may need to be targeted in vaccination campaigns.^6^ So, what are the risk factors for under-immunization in migrant communities, and how do we ensure better engagement and their inclusion in national vaccine plans?

## Multiple risk factors for under-immunization

Even before the pandemic, migrants were considered at risk of under-immunization, with lower levels of routine vaccine uptake and trust in vaccination compared with the general population. As well as potential reluctance to vaccinate, migrants face numerous, well-documented barriers to healthcare. Most European countries, for example, restrict access to healthcare and vaccination initiatives for certain migrant groups,^7^ which has undoubtedly resulted in lower engagement with mainstream services, despite the fact that ensuring high levels of coverage and equitable access are key priorities of the WHO’s European Vaccine Action Plan and the Sustainable Development Goals. Poor understanding of the host country’s health system, language and cultural barriers and poor doctor–patient relationships compound access issues. Some barriers may stem from longstanding structural inequities, as well as the fact that these communities tend to live in areas of higher deprivation. Ethnicity-related factors, including religion, upbringing and beliefs also influence immunization decisions.

Furthermore, these communities may be more susceptible to COVID-19 vaccine misinformation, particularly where language barriers and social exclusion contribute to a deficit of accurate information.^5^ In a series of participatory community workshops conducted with migrant community leaders in London,^8^ mistrust and unwillingness to vaccinate for COVID-19 were reported, with concerns raised about the extent of misleading COVID-19 vaccination information circulating in their communities via social media (including TikTok, Facebook and Whatsapp) and the perceived low representation of their communities in vaccine trials. One qualitative study of refugees and asylum seekers found they had a range of specific beliefs, including that COVID-19 is a ‘hoax’ or ‘Western disease’, or that it contains a microchip to control the population^5^; other shared views include that the vaccine will alter your DNA, may affect fertility or is not halal (permitted by Islam). Migrants and clinicians reported concerns that mistrust of the state and health system, stemming from historical events, data sharing policies and dissatisfaction with the initial handling of the pandemic, as well as low health literacy in migrant communities and widespread vaccine misinformation, have reinforced rumours and could negatively affect uptake rates.^5^^,^^8^ Low levels of COVID-19 in migrants’ countries of origin may also impact on their views on vaccination. Vaccination coverage data disaggregated by migrant status are, however, lacking in many health information systems, which has important consequences for understanding inequities.

## Experiences to date and policy response to vaccine roll-out

Many governments did not include migrants and ethnic minorities well in their national plans in the first wave of the pandemic,^4^ and it is crucial that we do not make the same mistakes with vaccine roll-out. In a rapid review to assess COVID-19 communications targeting migrants (June 2020), for example, only half of Council of Europe member states had translated information into at least one foreign language, while 6% (3/47) had translated information on testing or healthcare entitlements. None produced risk communications on disease prevention for refugee camps^9^; in the Greek camps hundreds of thousands of migrants were excluded from the national response.^10^ Other studies from Canada, Denmark and the US reported lags in translating official guidance into foreign languages and poor dissemination to, and hence access by, migrant communities; importantly, groups with lower language and literacy levels were also found to have lower testing rates.^11^^,^^12^ It is unsurprising, therefore, that preliminary monitoring data suggest that some of these communities are less likely to have been included in initial vaccine roll-out.

A key priority now must be to identify ways to engage with and deliver COVID-19 vaccination to marginalized migrants; an issue likely to remain salient beyond these immediate few months. There have been several positive developments. The European Centre for Disease Prevention and Control (ECDC) has classified migrants as potential target groups for vaccination campaigns and advised that overcrowded settings (e.g. reception centres, crowded housing and homeless shelters) are considered when deciding upon priorities for vaccination.^13^^,^^14^ WHO also identifies low-income migrant workers and irregular migrants as priority groups globally. The IOM (UN Migration Agency) has called on Member States to ensure all migrants, including undocumented ones, are included in national vaccine deployment plans. Some European governments have removed healthcare entitlement barriers to testing and vaccination for COVID-19 or stated that vaccines will be available irrespective of residence status (Spain, Netherlands, UK, France and Italy), whereas Germany has prioritized asylum seekers living in accommodation centres for vaccination. This alone is unlikely to encourage widespread uptake, but is undoubtedly an important first step in ensuring their inclusion, aligning with the principles of universal health coverage and health equity. At the same time, it is worth making a distinction between prioritization, and ensuring these communities have access akin to the rest of the population, with potential unintended consequences of prioritizing specific groups such as migrants who, in some contexts, may find it stigmatising and discriminatory; meanwhile, the native-born population may perceive this as undue benefit. Instead, we should advocate for these communities to be better considered within countries’ existing vaccine priority structures.

## Co-producing solutions based on the principles of inclusion and engagement

The next step is to ensure policy translates into practice, with population diversity better recognized by policymakers. This will require actively and meaningfully engaging with communities to understand their concerns or barriers to vaccination and working together to co-develop tailored approaches to encourage uptake and rebuild trust. Participatory approaches, community engagement and co-production, drawing on existing models of best practice and expertise, will be critical—strategies previously called for by WHO and ECDC to strengthen vaccination initiatives and, to some extent, reflected in WHO’s Tailoring Immunization Programmes framework, which aims to address barriers and leverage drivers of vaccination in populations with sub-optimal vaccination uptake. These approaches offer a collaborative model of research, where researchers, social scientists, community stakeholders and end-users work in partnership to identify a problem and co-produce knowledge, empowering communities to implement sustainable change. Recent studies and discourse have recommended improved community outreach and engagement through a variety of platforms, settings and messengers (e.g. opinion leaders and community champions), alongside greater consideration of the health, scientific and general literacy levels in specific subpopulations. Outreach efforts should also be complemented by longer-term strategies to support and encourage underserved members of the community to access health systems so they can be vaccinated.

Clear and concise written and visual resources for different language/literacy needs should be developed, ideally centrally, with community representatives actively guiding their development. They must be made available for local adaptation and distribution, with the best channels for dissemination decided by community members. Importantly, policymakers and researchers must be prepared to hand over power and responsibility to communities to lead inclusive, community-centred strategies for increasing COVID-19 vaccination uptake, while recognising the practical requirements and investment needed for co-production, and nurturing and sustaining these relationships and systems so that responses can be more efficiently mobilized in future public health crises.

**Figure 1:**
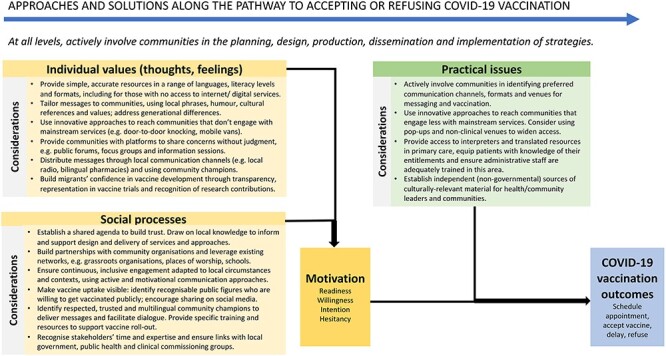
Approaches and solutions for COVID-19 vaccine roll-out in migrant communities. Footnote: Adapted from Brewer et al^15^

A WHO expert working group on behavioural and social drivers of vaccination developed the ‘Increasing Vaccination Model’ to establish the factors that influence vaccine uptake and pinpoint specific areas for intervention, considering (i) the motivation to get vaccinated (informed by feelings, emotions, social norms and processes) and (ii) practical issues (e.g. access/availability, convenience, cost and service satisfaction).^15^ The extent to which each driver contributes to low COVID-19 vaccination uptake in migrants remains to be explored. In Figure 1, we show how this model^15^ might be applied to strengthening COVID-19 vaccination uptake in these communities.

## Opportunities and next steps

Routine and timely vaccination of at-risk groups should be an urgent priority for any country that is serious about achieving control of COVID-19, and other vaccine-preventable diseases. As we seek to address the stark health inequalities exposed by this pandemic, it is essential that we urgently strengthen engagement and build trust with migrant communities and acquire a better understanding of how to support them. At the heart of this is ensuring they are more meaningfully included through more culturally competent health systems, where greater emphasis is placed on providing care to patients with diverse values and behaviours and tailoring delivery to meet patients’ social, cultural and linguistic needs.

**Key messages and recommendations**

- Ensure undocumented migrants, asylum seekers and other excluded migrant populations—including those residing in camps, reception and detention facilities—are meaningfully included in COVID-19 vaccination roll-out plans and supported to access health systems.
- Better consider migrants within the existing vaccine priority structure defined by individual countries, which may require specific tailored and targeted approaches considering their specific risk factors for under-immunization.
- Urgently conduct more research to explore key risk factors for under-immunization for COVID-19 in these communities, to assess the extent to which vaccine hesitancy and circulating misinformation is playing a role and to better elucidate physical and other structural barriers to vaccination.
- Actively involve communities in the planning, co-production, dissemination and implementation of tailored and targeted approaches to encourage widespread participation in COVID-19 vaccination programmes, and empower migrant and minority healthcare professionals within communities. It is vital that local government, public health teams and healthcare professionals establish trust with communities and build partnerships with local stakeholders through regular and meaningful engagement activities.
- Incentivise better recording of data or integrating core variables around ethnicity and migration for vaccine uptake into Health Information Systems, and strengthen the evidence-base to support innovative interventions and engagement around other vaccine-preventable diseases.
